# Examining Mental Health Outcomes of Intimate Partner Violence Among Female Survivors in Taiwan: A Population-Based Study

**DOI:** 10.1089/whr.2024.0203

**Published:** 2025-04-07

**Authors:** Ming-Yi Li, Hong-Xun Wang, Shin-Ting Yeh

**Affiliations:** ^1^ Department of Health Promotion and Health Education, National Taiwan Normal University, Taipei City, Taiwan.; ^2^ Institute of Clinical Nursing, National Yang Ming Chiao Tung University, Taipei City, Taiwan.; ^3^ Graduate Institute of Artificial Intelligence and Big Data in Healthcare, National Taipei University of Nursing and Health Sciences, Taipei City, Taiwan.

**Keywords:** intimate partner violence, mental health, women, population-based study, claims data

## Abstract

**Objective:**

Intimate partner violence (IPV) significantly affects women’s health, but the lack of control groups in past studies hinders identifying high-risk populations and establishing evidence-based interventions. This study examines the link between IPV and women’s mental health outcomes.

**Methods:**

A nationwide database was used in this case–control study. The study targeted adult women aged 18–64 in 2019 and employed a case–control study design with a total sample size of 71,512 participants. Data were sourced from the Ministry of Health enrollment files, cause of death statistics, and outpatient and inpatient claims data. Chi-square tests were used to analyze the association between IPV with mental health outcomes and personal characteristics. Additionally, conditional logistic regression models were applied to investigate the impact of IPV on women’s mental health outcomes.

**Results:**

Compared with nonvictims, women who experienced IPV had significantly lower socioeconomic status and were at higher risk for various mental health outcomes. These included bipolar disorder (adjusted odds ratio [OR] = 5.91, 95% confidence interval [CI]: 4.16–8.38), alcohol and substance abuse (adjusted OR = 4.84, 95% CI: 2.88–8.14), depression (adjusted OR = 4.67, 95% CI: 3.91–5.58), schizophrenia (adjusted OR = 2.37, 95% CI: 1.80–3.12), and anxiety (adjusted OR = 2.36, 95% CI: 1.98–2.81).

**Conclusions:**

This study identified several mental disorders highly associated with IPV among adult women, with particular emphasis on bipolar disorder, alcohol and substance abuse, and depression. Insights into high-risk mental health disorders will help clinical staff be alert to IPV and provide a reference for policy planning of IPV counseling and intervention.

## Introduction 

Intimate partner violence (IPV) is a significant form of domestic violence, defined by the World Health Organization (WHO) as physical, sexual, or psychological harm inflicted by a current or former partner.^1^ This type of violence is associated with various health-risk behaviors and has profound effects on the physical and mental health of women, men, and children, leading to substantial economic and social costs.^2,3^ IPV is recognized as a global public health and social issue that has reached epidemic proportions. Although both men and women are affected, women experience IPV more frequently and with greater severity. According to WHO estimates, in 2018, 26% of ever-married or cohabiting women aged 15 years or older worldwide had experienced IPV.^1^ Studies indicate that women are 1.3 times more likely to experience IPV in comparison with men.^4,5^ In the United States, 43.6% of women experienced some form of contact sexual violence in their lifetime, and approximately 20% women in the United States reported completed or attempted rape at some point in their lifetime.^6^ A majority of female victims of completed or attempted rape first experienced such victimization early in life, with 81.3% reporting that it first occurred prior to age 25, and 43.2% reported that it first occurred prior to age 18.^6^ In Taiwan, official statistics indicate a yearly increase in reported domestic violence cases, with IPV accounting for nearly half of all reported.^7^ A 2020 survey report in Taiwan revealed that the lifetime prevalence of IPV among women aged 18–74 was 17.06%, highlighting the urgent need to address IPV among women.^8^

The impact of IPV on female survivors includes direct physical injuries as well as indirect health issues resulting from prolonged stress.^9^ Research has confirmed strong associations between IPV and mental health problems, including depression, anxiety, and lowered self-esteem, as well as heightened risks for conditions such as anxiety disorders, depression, and alcohol abuse.^10,11^ Regarding depression, IPV experiences are considered significant predictors of depression, with the severity of violence correlating with the exacerbation of depressive symptoms.^12^ Women who have experienced IPV have a 2.43–3.9 times higher risk of developing depression compared with non-abused women.^13–15^ Additionally, women at high risk of IPV are 2.10–3.21 times more likely to exhibit depressive symptoms compared with those at low risk.^11,16^ For anxiety disorders, IPV survivors, who often live in persistent fear, are 1.91–3.9 times more likely to develop anxiety disorders than non-abused women.^11,13,17,18^ Studies also indicate that preexisting mental health problems can increase IPV risk.^19,20^ For instance, women with severe mental illnesses, such as bipolar disorder and schizophrenia, are 2.13–2.6 times more likely to experience IPV than women without these conditions.^13,21^ IPV is also a significant predictor of alcohol and substance abuse, as survivors may turn to substances or excessive drinking as coping mechanisms for emotional distress and stress. Women exposed to IPV face a 1.47–2.88 times higher risk of alcohol abuse and a 1.23–2.50 times higher risk of substance abuse.^13,21^

Over a quarter of women worldwide experience IPV in their lifetime, with women comprising up to 80% of IPV survivors in Taiwan, highlighting the critical need to examine IPV’s effects on women. However, past research has primarily focused on abused women without comparable control groups, limiting the ability to infer the prevalence or high-risk associations of common mental health disorders among IPV survivors. This gap affects the definition and empirical basis for identifying high-risk populations for IPV. This study aims to analyze the relationship between IPV and women’s mental health status using a national secondary database. It will compare mental health outcomes between women who have experienced IPV and those who have not, providing empirical evidence to inform relevant policies and intervention strategies.

## Methods

### Research design

This study employs a case–control design to investigate the impact of IPV on the mental health of adult women. The study population includes adult women aged 18–64 nationwide in 2019, with IPV-experienced women serving as the case group. A 1:1 frequency matching based on 3-year age-groups was conducted to select non-abused women as the control group. The enrollment period for this study was from January 1, 2019, to December 31, 2019, while the overall study period extended from January 1, 2019, to December 31, 2020. This study has received approval from the Institutional Review Board of National Yang Ming Chiao Tung University (IRB number YM111102E).

### Measures

The data for this study were obtained from the Health and Welfare Data Science Center, Ministry of Health and Welfare. The databases used include the Domestic Violence Reporting Database, National Health Insurance Enrollment Files, Mortality Registry, and National Health Insurance Outpatient and Inpatient Claims Data. The Domestic Violence Reporting Database provides information on victims and perpetrators of domestic violence each year, with IPV cases identified through the “Relationship of Parties” field. The National Health Insurance Enrollment Files include demographic characteristics of the entire population; this study used variables such as gender and birth date to define study subjects and individual characteristics and performed a 1:1 frequency match by age to establish a control group. The Mortality Registry was used to exclude cases that died during the study period. Additionally, the National Health Insurance Claims Data was utilized to extract primary and secondary diagnostic codes for outpatient and inpatient visits within one year following a report, which served to define mental health conditions among the cases. Integrating these datasets enables a comprehensive analysis of the impact of IPV on women’s mental health.

**FIG. 1.**
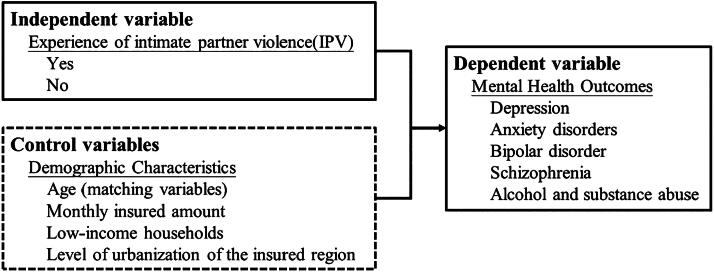
Research framework.

The independent variable in this study is “experience of intimate partner violence (IPV).” IPV cases were identified using the “Relationship of Parties” field in the 2019 Domestic Violence Reporting Database, with codes “A (Currently Married),” “B (Divorced),” “C (Cohabiting Partner),” and “D (Former Cohabiting Partner)” categorized as IPV cases. To ensure the inclusion of new cases, individuals with domestic violence reports in 2018 were excluded. Only newly reported cases from 2019 were included as study subjects. If an individual had multiple reports in 2019, the first report date was used as the starting point for follow-up (Fig. 1).

The dependent variable in this study is the occurrence of various mental health disorders, with the selection of disorder types based on definitions commonly associated with IPV survivors in the literature. This study utilized the National Health Insurance outpatient and inpatient claims data to extract primary and secondary diagnostic codes for health care visits within 1 year following the initial report. A case was defined as having a specific mental health disorder if the diagnosis appeared at least twice in outpatient claims or at least once in inpatient claims.^22^ This study classified mental health disorders based on the International Classification of Diseases, 10th Revision (ICD-10-CM). The disorders examined included depression (ICD-10-CM: F32–F39),^23^ anxiety disorders (ICD-10-CM: F40–F42, F44, F45, F48),^23^ bipolar disorder (ICD-10-CM: F30, F31),^23^ schizophrenia (ICD-10-CM: F20–F29),^23^ and alcohol and substance abuse (ICD-10-CM: F10–F16, F18, F19).^24^ These disorders were included as the focus of this study (Fig. 1).

For control variables, definitions were derived using various fields from the National Health Insurance Enrollment Files. Age was calculated by subtracting the birth date from the midpoint of 2019 (July 1, 2019) and categorized into five groups. Monthly insured amount was classified into three categories based on the 2019 Family Income and Expenditure Survey published by the Directorate General of Budget, Accounting and Statistics: low (≤$28,860), medium ($28,861–$43,642), and high (>$43,642).^25^ Low-income households were identified based on insured unit type codes 51 or 52.^26^ The level of urbanization of the insured region was determined using the insured unit’s regional code and categorized into seven levels, which were further consolidated into three categories: high (highly urbanized towns and moderately urbanized towns), medium (emerging towns and general townships), and low (aging towns, agricultural towns, and remote townships)^27,28^ (Fig. 1).

### Analysis

This study utilized SAS 9.4 software for secondary data analysis. Descriptive statistics were used to present the demographic characteristics and distribution of mental health disorders among participants with and without IPV, expressed as frequencies and percentages. For inferential statistics, bivariate analysis was conducted using chi-square tests to examine the association between IPV with mental health disorders and demographic. Multivariate analysis employed a conditional logistic regression model to investigate the impact of IPV on different types of mental health disorders among women, adjusting for demographic characteristics. This approach provides a deeper understanding of the potential effects of IPV on women’s health and offers empirical evidence to inform related policies.

## Results

This study analyzed women aged 18–64 who were reported as victims of IPV in 2019. Considering the differences in age composition between the IPV and non-IPV groups, the case group consisted of women who experienced IPV, and a control group was formed through 1:1 frequency matching by age (in 3-year intervals), resulting in 35,756 participants in each group (Table 1). The study aimed to compare the distribution of mental health disorders and personal characteristics between women who experienced IPV and those who did not.

**Table 1.** Demographic Characteristics of Women with and Without Intimate Partner Violence Experiences Before Matching in 2019

Variables	Non-IPV		IPV		*p*-Value
	Sample size (*n*)	Percentage (%)	Sample size (*n*)	Percentage (%)	
Total	8,174,303	100.00	35,756	100.00	
Age					<0.0001
18–24	988,915	12.10	2,699	7.55	
25–29	763,783	9.43	3,572	9.99	
30–39	1,756,589	21.49	12,271	34.32	
40–49	1,909,328	23.36	10,374	29.01	
50–64	2,755,688	33.71	6,840	19.13	
Monthly insured amount					<0.0001
Low (≤$28,860)	4,794,356	58.65	26,768	74.86	
Medium($28,861–$43,642)	1,573,829	19.25	4,652	13.01	
High (>$43,642)	1,806,118	22.10	4,336	12.13	
Low-income households					<0.0001
None	8,096,361	99.05	34,375	96.14	
Yes	77,942	0.95	1,381	3.86	
Level of urbanization of the insured region					<0.0001
High	5,085,844	62.22	19,955	58.40	
Medium	2,240,885	27.41	11,087	32.45	
Low	459,750	5.62	3,129	9.16	
Missing	387,824	4.74	1,585	4.43	

IPV, intimate partner violence.

In terms of demographic characteristics, the majority of women who experienced IPV were aged 30–39 years (34.32%) and 40–49 years (29.01%). Additionally, 74.86% of IPV survivors had low insured amounts, and 3.86% were from low-income households, both higher than the corresponding percentages among non-IPV survivors (57.61% and 0.98%, respectively). Regarding the level of urbanization, the proportion of IPV survivors residing in highly urbanized (32.45%) and low-urbanized (9.16%) areas was also higher than that of non-IPV survivors (28.99% and 5.56%, respectively) (Table 2).

**Table 2.** Demographic Characteristics and Mental Health Outcomes of Women with and Without Intimate Partner Violence Experiences After Matching in 2019

Variables	Non-IPV		IPV		*p*-Value
	Sample size (*n*)	Percentage (%)	Sample size (*n*)	Percentage (%)	
Total	35,756	100.00	35,756	100.00	
Age					0.9512
18–24	2,722	7.61	2,699	7.55	
25–29	3,549	9.93	3,572	9.99	
30–39	12,188	34.09	12,271	34.32	
40–49	10,404	29.10	10,374	29.01	
50–64	6,893	19.28	6,840	19.13	
Monthly insured amount					<0.0001
Low (≤$28,860)	20,599	57.61	26,768	74.86	
Medium($28,861–$43,642)	7,196	20.13	4,652	13.01	
High (>$43,642)	7,961	22.26	4,336	12.13	
Low-income households					<0.0001
None	35,405	99.02	34,375	96.14	
Yes	351	0.98	1,381	3.86	
Level of urbanization of the insured region					<0.0001
High	22,207	65.45	19,955	58.40	
Medium	9,835	28.99	11,087	32.45	
Low	1,886	5.56	3,129	9.16	
Missing	1,828	5.11	1,585	4.43	
Mental Health Outcomes					
Depression	154	0.43	803	2.25	<0.0001
Anxiety disorders	192	0.54	489	1.37	<0.0001
Bipolar disorder	38	0.11	251	0.70	<0.0001
Schizophrenia	73	0.20	215	0.60	<0.0001
Alcohol and substance abuse	17	0.05	106	0.30	<0.0001

IPV, intimate partner violence.

In terms of mental health disorders, IPV survivors had higher prevalence rates compared with non-IPV survivors for depression (2.25% vs. 0.43%), anxiety disorders (1.37% vs. 0.54%), bipolar disorder (0.70% vs. 0.11%), schizophrenia (0.60% vs. 0.20%), and alcohol and substance abuse (0.30% vs. 0.05%). Chi-square tests were conducted to examine the associations between IPV experience with mental health disorders and demographic characteristics. The results indicated significant differences between IPV experience and insured amount, low-income household status, urbanization level, and all types of mental health disorders (Table 2).

This study utilized conditional logistic regression analysis to assess the impact of IPV experience on various mental health disorders (Table 3). In unadjusted models, the results revealed that women with a history of IPV exhibited a significantly higher risk of various psychological disorders compared with those without such experiences. A significant difference was found between the two groups in the risk of developing depression (crude odds ratio [OR] = 5.31, 95% confidence interval [CI]: 4.47–6.31, *p* < 0.0001), anxiety disorders (crude OR = 2.57, 95% CI: 2.17–3.04, *p* < 0.0001), bipolar disorder (crude OR = 6.65, 95% CI: 4.72–9.35, *p* < 0.0001), schizophrenia (crude OR = 2.96, 95% CI: 2.27–3.86, *p* < 0.0001), and alcohol and substance abuse (crude OR = 6.25, 95% CI: 3.75–10.43, *p* < 0.0001) (Table 3).

**Table 3.** Using Conditional Logistic Regression to Exploring the Association Between Intimate Partner Violence and Mental Health Outcomes Among Women in 2019

Variables	Univariable and multivariable analyses						
	Model 1^a^			Model 2^b^			
	Crude ORs	(95% CI)	*p*-Value	Adjusted ORs	(95% CI)	*p*-Value	Index (c)
Mental health outcomes (ref group: non)							
Depression	5.31	(4.47–6.31)	<0.0001	4.67	(3.91–5.58)	<0.0001	0.672
Anxiety disorders	2.57	(2.17–3.04)	<0.0001	2.36	(1.98–2.81)	<0.0001	0.610
Bipolar disorder	6.65	(4.72–9.35)	<0.0001	5.91	(4.16–8.38)	<0.0001	0.748
Schizophrenia	2.96	(2.27–3.86)	<0.0001	2.37	(1.80–3.12)	<0.0001	0.624
Alcohol and substance abuse	6.25	(3.75–10.43)	<0.0001	4.84	(2.88–8.14)	<0.0001	0.681

^a^ Analysis by conditional logistic regression.

^b^ Analysis by multivariate conditional logistic regression, and the model was adjusted for age, monthly insured amount, low-income households, and level of urbanization of the insured region to identify the mental health outcomes associated with IPV.

CI, confidence interval; OR, odds ratio; IPV, intimate partner violence.

After adjusting for personal characteristics, the analysis revealed that women with experiences of violence still had a significantly higher risk of developing depression (adjusted OR = 4.67, 95% CI: 3.91–5.58, *p* < 0.0001), anxiety disorders (adjusted OR = 2.36, 95% CI: 1.98–2.81, *p* < 0.0001), bipolar disorder (adjusted OR = 5.91, 95% CI: 4.16–8.38, *p* < 0.0001), schizophrenia (adjusted OR = 2.37, 95% CI: 1.80–3.12, *p* < 0.0001), and alcohol and substance abuse (adjusted OR = 4.84, 95% CI: 2.88–8.14, *p* < 0.0001) compared with those without such experiences. A significant difference was found between the two groups, although the risk of these disorders decreased compared with the results without controlling for individual characteristics, indicating that individual traits play an important role in the likelihood of developing psychological disorders. This study’s entire conditional logistic regression model demonstrated validity based on the Testing Global Null Hypothesis. The likelihood ratio *p* value of all full models is ≤0.0001. The strength of the entire model was assessed using the area under the ROC curve, determined by the trapezoidal rule, and the value was estimated by the concordance index (c), which is reported in the “Association of Predicted Probabilities and Observed Responses” table. The concordance index (c) of all full models is 0.672 (depression), 0.610 (anxiety disorders), 0.748 (bipolar disorder), 0.624 (schizophrenia), and 0.681 (alcohol and substance abuse). The findings align with the expectations outlined in the research hypothesis (Table 3).

## Discussion

This study aimed to explore the association between experiences of IPV and women’s mental health outcomes. The findings revealed that IPV experiences significantly increase the risk of various mental health disorders, including depression, anxiety disorders, bipolar disorder, schizophrenia, and alcohol and substance abuse. These results are consistent with previous studies.^11,13–15^ The findings underscore the substantial impact of IPV on women’s mental health and highlight the urgent need for more effective support and intervention strategies for this population.

The results of this study indicate that women who have experienced IPV are at a significantly higher risk of developing depression compared with those without IPV experiences, consistent with previous literature.^13,29,30^ Research suggests that perpetrators often employ behaviors such as belittlement and humiliation to erode victims’ self-esteem while also restricting their social activities, leading to isolation. Both reduced self-worth and a lack of social support are critical factors that increase the risk of depression^31^ Additionally, studies on IPV survivors in Taiwan reveal that, despite protective orders against perpetrators, victims may still face threats or verbal abuse. Furthermore, IPV is often a sustained pattern of behavior rather than a single incident. As a result, survivors not only endure violence but also grapple with household responsibilities and societal role expectations, compounding their stress and further elevating the risk of depression.^32,33^

This study confirms that women who have experienced IPV are at a significantly higher risk of developing anxiety compared with those without IPV experiences. According to research by Bacchus et al.,^34^ IPV has profound negative impacts on women’s emotional, psychological, and physical well-being. Women subjected to frequent physical or emotional violence often exhibit persistent stress responses, making them more susceptible to developing mental health conditions such as anxiety. This stress response is linked to areas of the brain that regulate emotions, and prolonged tension and fear can heighten sensitivity to perceived threats in daily life, increasing the risk of anxiety disorders. Further research by Clemente-Teixeira et al. highlights that women who experience IPV may suffer from diminished self-esteem and a lowered sense of self-worth, leading to feelings of insecurity in their environment and exacerbating anxiety symptoms. This psychological trauma can also affect physical health, causing sleep disturbances and weakened immune function, which further worsens anxiety symptoms.^35^

The findings of this study demonstrate that women who have experienced IPV are at a significantly higher risk of developing bipolar disorder, consistent with previous research. This may be attributed to the disruptive effects of violence on emotional stability and stress response, which increase the likelihood of mood disorders such as bipolar disorder. Experiences of violence often induce extreme emotional fluctuations, resembling the hallmark features of bipolar disorder, including cyclical changes between elevated and depressed moods.^21,34,36^ Additionally, related studies suggest that IPV interferes with emotional regulation, leading to chronic stress and trauma symptoms, both of which are potential triggers for bipolar disorder. The trauma associated with violence can have long-term effects on the brain’s stress response system, impairing emotional regulation and thereby increasing the risk of developing bipolar disorder.^37^

Women who experience IPV face an increased risk of developing schizophrenia and other severe mental illnesses. This may be due to the heightened psychological stress within abusive relationships, which exacerbates mental health issues such as anxiety, depression, and post-traumatic stress disorder. These conditions can impact brain function and cognitive abilities, ultimately leading to more severe psychotic symptoms.^13,38^ Furthermore, the long-term negative effects of physical or emotional violence on the brain may disrupt the regulation of dopamine and other neurotransmitters, resulting in neurochemical changes that could contribute to the onset of schizophrenia and related psychiatric disorders.^23^ IPV is also often accompanied by unhealthy behaviors, such as alcohol consumption or substance abuse, which may further compound the risk of mental illnesses. These behaviors can trap women in abusive relationships within a vicious cycle, significantly increasing the likelihood of developing schizophrenia.^13,38^

The findings of this study indicate that women who experience IPV are at a higher risk of alcohol and substance abuse. This is primarily due to the psychological and physical trauma endured in abusive relationships, which may prompt victims to engage in self-medicating behaviors. They may turn to alcohol or drugs as a means of alleviating anxiety, depression, post-traumatic stress, and other negative emotions, thereby attempting to regulate their mood.^39,40^ Additionally, IPV may result in physical injuries, such as head or neck trauma, which are closely linked to mental health issues and further increase the risk of substance abuse.^40^ Factors such as a lack of psychological support, insufficient resources, and economic hardship can also drive IPV survivors to rely on alcohol or drugs to cope with the stresses of daily life.^40^ These findings highlight the multiple challenges faced by women in abusive relationships and the coping strategies they adopt in response to psychological and physical trauma. Providing appropriate support and resources is critical to helping these women break free from the vicious cycle of violence and substance dependence.

This study found that low-income women face a significantly higher prevalence of IPV, consistent with prior research.^41,42^ Low income impacts IPV through pathways such as financial stress, economic dependence, and the intersection of poverty with other vulnerabilities. Both objective and subjective economic hardships increase IPV risk,^43^ with higher frequencies observed in low- and middle-income countries^44^ and disadvantaged communities.^43^ Economic abuse, present in 99% of domestic violence cases, often traps women in abusive relationships due to dependence.^45^ Additionally, poverty, combined with chronic stressors such as poor housing and limited resources, exacerbates IPV risk, as do interrelated factors such as low education and unemployment.^45,46^

This study found that women in low-urbanization areas face a higher risk of IPV than those in urban regions. Research in Iowa, USA, showed IPV rates of 22.5% in small rural areas, 17.9% in remote areas, and 15.5% in urban areas, with rural women experiencing more severe physical abuse.^42^ Remote areas may conceal abusive behaviors, as perpetrators are more likely to reside there.^47,48^ These regions often lack IPV prevention resources, such as medical assistance and shelters, increasing the risk.^42^ Sociocultural factors, including conservative beliefs and male-dominated family structures, can normalize violence and hinder victims from leaving abusive relationships.^42^

This study highlights the profound impact of IPV on women’s mental health and the importance of addressing it through effective health care policies. According to the WHO,^49^ many IPV survivors face inadequate care, with two-thirds of women in mental health services having experienced IPV. Some services are ineffective or harmful, causing re-traumatization. Raising awareness of the IPV–mental health connection and training health care providers to recognize and address IPV are essential. Services must be sensitive, inclusive, and comprehensive, offering safety planning, mental health support, legal assistance, and follow-up care. Such integrated approaches can improve survivors’ mental health, promote early intervention, and reduce IPV prevalence and impact.

This study has two major strengths: (1) It utilizes a nationwide population database and employs quantitative research methods to investigate the mental health outcomes following IPV. This approach addresses the limitations of previous literature, which predominantly relied on qualitative research and was unable to clearly identify high-risk mental health conditions associated with IPV. (2) The data used in this study were sourced from the Health and Welfare Data Science Center of the Ministry of Health and Welfare, including medical claims and domestic violence reporting data. This approach minimizes the cognitive bias issues that may arise from self-reported questionnaires or measurement scales when assessing IPV experiences and disease types. However, the study also has two limitations: (1) The definition of IPV is based on domestic violence reporting data. While using official reports ensures more precise identification of IPV cases, the existence of unreported cases may result in the study not fully encompassing all IPV instances. (2) This study examines the association between IPV in 2019 and women’s mental health conditions, with mental health disorders defined based on medical claims data within 1 year after the reporting date. The analysis does not exclude preexisting mental health conditions prior to the reporting date. Future research is recommended to further clarify the causal relationship between IPV experiences and mental health disorders and to explore the long-term impacts of IPV on women’s mental health.

## Conclusions

The findings of this study reveal that women who experience IPV have a significantly higher risk of developing various mental health disorders compared with non-abused women. Therefore, it is recommended that health care institutions enhance professionals’ sensitivity to identifying IPV, particularly among women with high-risk mental health conditions. This would help to avoid overlooking help-seeking behaviors and the psychological issues that often accompany repeated medical visits linked to IPV. Additionally, current government-provided therapeutic services and financial subsidies for domestic violence survivors primarily focus on issues such as depression, bipolar disorder, and self-identity. However, there is a lack of specialized services addressing severe mental illnesses such as schizophrenia and substance abuse, including alcohol and drug dependency. It is recommended that future care services for IPV survivors become more diversified to more effectively assist victims in overcoming their challenges and fostering psychological recovery. For future research, it is suggested to further explore the causal relationship between IPV and mental health disorders and to analyze the long-term effects of IPV on the psychological well-being of women. Such research would provide empirical evidence for more targeted policy planning, ultimately improving the quality of life and mental health of IPV survivors.

## Abbreviations Used

CI: confidence interval
ICD-10-CM: International Classification of Diseases, 10th Revision
IPV: Intimate partner violence
OR: odds ratio
WHO: World Health Organization

## References

[1] Organization WH. Violence Against Women Prevalence Estimates, 2018: Global, Regional and National Prevalence Estimates for Intimate Partner Violence Against Women and Global and Regional Prevalence Estimates for Non-Partner Sexual Violence Against Women. World Health Organization; 2021.

[2] Dicola D, Spaar E. Intimate partner violence. Am Fam Physician 2016;94(8):646–651. 27929227

[3] Wathen CN, MacGregor JCD, Hammerton J, et al.; PreVAiL Research Network. Priorities for research in child maltreatment, intimate partner violence and resilience to violence exposures: Results of an international Delphi consensus development process. BMC Public Health 2012;12:684. 22908894 10.1186/1471-2458-12-684 PMC3490760

[4] Beydoun HA, Beydoun MA, Kaufman JS, et al. Intimate partner violence against adult women and its association with major depressive disorder, depressive symptoms and postpartum depression: A systematic review and meta-analysis. Soc Sci Med 2012;75(6):959–975. 22694991 10.1016/j.socscimed.2012.04.025 PMC3537499

[5] Coker AL, Davis KE, Arias I, et al. Physical and mental health effects of intimate partner violence for men and women. Am J Prev Med 2002;23(4):260–268. 12406480 10.1016/s0749-3797(02)00514-7

[6] Smith SG, Zhang X, Basile KC, et al. The National Intimate Partner and Sexual Violence Survey: 2015 data brief–updated release. 2018. 10.1016/j.amepre.2018.01.014 PMC6007810 29449134

[7] Welfare MoHa. Statistical Report on Domestic Violence Cases 2021. Available from: https://dep.mohw.gov.tw/dops/lp-1303-105-xCat-cat01.html

[8] Pan S-M. 2020 Statistical Survey on Intimate Partner Violence Against Women in Taiwan (M09C7266). Ministry of Health and Welfare: Taipei; 2021.

[9] Chisholm CA, Bullock L, Ferguson II J. Intimate partner violence and pregnancy: Screening and intervention. Am J Obstet Gynecol 2017;217(2):145–149. 28551447 10.1016/j.ajog.2017.05.043

[10] Campbell JC. Health consequences of intimate partner violence. Lancet 2002;359(9314):1331–1336. 11965295 10.1016/S0140-6736(02)08336-8

[11] Makaroun LK, Brignone E, Rosland A-M, et al. Association of health conditions and health service utilization with intimate partner violence identified via routine screening among middle-aged and older women. JAMA Netw Open 2020;3(4):e203138-e. 32315066 10.1001/jamanetworkopen.2020.3138 PMC7175082

[12] Dillon G, Hussain R, Loxton D, et al. Mental and physical health and intimate partner violence against women: A review of the literature. Int J Family Med 2013;2013(1):313909. 23431441 10.1155/2013/313909 PMC3566605

[13] Chandan JS, Thomas T, Bradbury-Jones C, et al. Female survivors of intimate partner violence and risk of depression, anxiety and serious mental illness. Br J Psychiatry 2020;217(4):562–567. 31171045 10.1192/bjp.2019.124

[14] Rodriguez MA, Heilemann MV, Fielder E, et al. Intimate partner violence, depression, and PTSD among pregnant Latina women. Ann Fam Med 2008;6(1):44–52. 18195314 10.1370/afm.743 PMC2203409

[15] Vung ND, Ostergren P-O, Krantz G. Intimate partner violence against women, health effects and health care seeking in rural Vietnam. Eur J Public Health 2009;19(2):178–182. 19131396 10.1093/eurpub/ckn136

[16] Iverson KM, Vogt D, Dichter ME, et al. Intimate partner violence and current mental health needs among female veterans. J Am Board Fam Med 2015;28(6):772–776. 26546653 10.3122/jabfm.2015.06.150154

[17] Brown SJ, Mensah F, Giallo R, et al. Intimate partner violence and maternal mental health ten years after a first birth: An Australian prospective cohort study of first-time mothers. J Affect Disord 2020;262:247–257. 31732279 10.1016/j.jad.2019.11.015

[18] Choudhary E, Smith M, Bossarte RM. Depression, anxiety, and symptom profiles among female and male victims of sexual violence. Am J Mens Health 2012;6(1):28–36. 22105064 10.1177/1557988311414045

[19] González Cases J, Polo Usaola C, González Aguado F, et al. Prevalence and characteristics of intimate partner violence against women with severe mental illness: A prevalence study in Spain. Community Ment Health J 2014;50(7):841–847. 24474531 10.1007/s10597-014-9703-1

[20] Suparare L, Watson SJ, Binns R, et al. Is intimate partner violence more common in pregnant women with severe mental illness? A retrospective study. Int J Soc Psychiatry 2020;66(3):225–231. 31902275 10.1177/0020764019897286

[21] Okuda M, Olfson M, Hasin D, et al. Mental health of victims of intimate partner violence: Results from a national epidemiologic survey. Psychiatr Serv 2011;62(8):959–962. 21807838 10.1176/appi.ps.62.8.959 PMC3204944

[22] Lin I-P, Wu S-C, Huang S-T. Continuity of care and avoidable hospitalizations for chronic obstructive pulmonary disease (COPD). J Am Board Fam Med 2015;28(2):222–230. 25748763 10.3122/jabfm.2015.02.140141

[23] Yu R, Nevado-Holgado AJ, Molero Y, et al. Mental disorders and intimate partner violence perpetrated by men towards women: A Swedish population-based longitudinal study. PLoS Med 2019;16(12):e1002995. 31846461 10.1371/journal.pmed.1002995 PMC6917212

[24] Kishton R, Sinko L, Ortiz R, et al. Describing the health status of women experiencing violence or abuse: An observational study using claims data. J Prim Care Community Health 2022;13:21501319221074121. 35345928 10.1177/21501319221074121 PMC8968984

[25] Directorate-General of Budget AaSD. National Statistical Bulletin 2022. Available from: https://www.dgbas.gov.tw/public/Data/292916010CA7EAUC2.pdf

[26] Szu-Chen Lin M-CK, Tang S-C, Chen C-Y, et al. Prevalence of and predictors for frequent utilization of emergency department of older adults. Journal of Healthcare Management 2018;19(1):43–62.

[27] Liu CY, Huang YT, Chuang Y-L, et al. Incorporating development stratification of Taiwan townships into sampling design of large scale health interview survey. Journal of Health Management 2006;4(1):1–22.

[28] Yi-Chen Chiang L-YW. The employment status and related factors of Taiwanese workers after acute myocardial infarction. Taiwan Journal of Publich Health/Taiwan Gong Gong Wei Sheng Za Zhi 2022;41(6).

[29] Devries KM, Mak JY, Bacchus LJ, et al. Intimate partner violence and incident depressive symptoms and suicide attempts: A systematic review of longitudinal studies. PLoS Med 2013;10(5):e1001439. 23671407 10.1371/journal.pmed.1001439 PMC3646718

[30] White SJ, Sin J, Sweeney A, et al. Global prevalence and mental health outcomes of intimate partner violence among women: A systematic review and meta-analysis. Trauma Violence Abuse 2024;25(1):494–511. 36825800 10.1177/15248380231155529 PMC10666489

[31] Wang P-L. The development and validation of Taiwan Intimate Partner Violence Danger Assessment (TIPVDA). Social Policy & Social Work 2012;16(1):1–58.

[32] Hsieh H-F. The Factors Related to Depressive Symptoms in Women Who Suffering from Domestic Violence. National Cheng Kung University: Tainan; 2008.

[33] Hsieh H-F, Shu B-C. Factors associated with depressive symptoms in female victims of intimate partner violence in Southern Taiwan. J Nurs Res 2019;27(4):e33. 30664041 10.1097/jnr.0000000000000303 PMC6641094

[34] Bacchus LJ, Ranganathan M, Watts C, et al. Recent intimate partner violence against women and health: A systematic review and meta-analysis of cohort studies. BMJ Open 2018;8(7):e019995. 10.1136/bmjopen-2017-019995 PMC6067339 30056376

[35] Clemente-Teixeira M, Magalhães T, Barrocas J, et al. Health outcomes in women victims of intimate partner violence: A 20-year real-world study. Int J Environ Res Public Health 2022;19(24):17035. 36554916 10.3390/ijerph192417035 PMC9779804

[36] Mahase E. Women who experience domestic abuse are three times as likely to develop mental illness. British Medical Journal Publishing Group 2019. 10.1136/bmj.l4126 31175081

[37] Trevillion K, Oram S, Feder G, et al. Experiences of domestic violence and mental disorders: A systematic review and meta-analysis. PLoS One 2012;7(12):e51740. 23300562 10.1371/journal.pone.0051740 PMC3530507

[38] Scoglio AAJ, Zhu Y, Lawn RB, et al. Intimate partner violence, mental health symptoms, and modifiable health factors in women during the COVID-19 pandemic in the US. JAMA Netw Open 2023;6(3):e232977–e. 36917107 10.1001/jamanetworkopen.2023.2977 PMC10015312

[39] Gil-Gonzalez D, Vives-Cases C, Alvarez-Dardet C, et al. Alcohol and intimate partner violence: Do we have enough information to act? Eur J Public Health 2006;16(3):279–285. 16476682 10.1093/eurpub/ckl016

[40] Mehr JB, Bennett ER, Price JL, et al. Intimate partner violence, substance use, and health comorbidities among women: A narrative review. Front Psychol 2022;13:1028375. 36778165 10.3389/fpsyg.2022.1028375 PMC9912846

[41] Gustafsson HC, Cox MJ, Investigators FLPK, Family Life Project Key Investigators. Intimate partner violence in rural low-income families: Correlates and change in prevalence over the first 5 years of a child’s life. J Fam Violence 2016;31(1):49–60. 26709334 10.1007/s10896-015-9760-4 PMC4687491

[42] Peek-Asa C, Wallis A, Harland K, et al. Rural disparity in domestic violence prevalence and access to resources. J Womens Health (Larchmt) 2011;20(11):1743–1749. 21919777 10.1089/jwh.2011.2891 PMC3216064

[43] Benson ML, Fox GL. When violence hits home: How economics and neighbourhood play a role, research in brief. 2004.

[44] Raj A. Is intimate partner violence declining in low-income and middle-income countries? Lancet Glob Health 2023;11(12):e1828–e9. 37973324 10.1016/S2214-109X(23)00471-0

[45] Children CfAWaT. Socioeconomic Risk Factors for Domestic and Intimate Partner Violence. 2024. Available from: https://www.cawc.org/news/socioeconomic-risk-factors-for-domestic-and-intimate-partner-violence/

[46] Hill TD, Mossakowski KN, Angel RJ. Relationship violence and psychological distress among low-income urban women. J Urban Health 2007;84(4):537–551. 17457676 10.1007/s11524-007-9187-1 PMC2219565

[47] Murty SA, Peek-Asa C, Zwerling C, et al. Physical and emotional partner abuse reported by men and women in a rural community. Am J Public Health 2003;93(7):1073–1075. 12835183 10.2105/ajph.93.7.1073 PMC1447907

[48] Riddell T, Ford-Gilboe M, Leipert B. Strategies used by rural women to stop, avoid, or escape from intimate partner violence. Health Care Women Int 2009;30(1–2):134–159. 19116826 10.1080/07399330802523774

[49] World Health Organization. Preventing intimate partner violence improves mental health. 2022. Available from: https://www.who.int/news/item/06-10-2022-preventing-intimate-partner-violence-improves-mental-health

